# Supplementary material for: Modeling glioblastoma heterogeneity as a dynamic network of cell states
Source: Mol Syst Biol. 2021 Sep 16;17(9):e10105. doi: 10.15252/msb.202010105 (PMC8444284; doi:10.15252/msb.202010105)
Supplement: Supplementary file 6 — Source Data for Figure 5 [file MSB-17-e10105-s004.zip › Figure5A_sourcedata/GSEA_3071/nosigdb_stateII.GseaPreranked.1622795151171/RICHARDS_NOWAKOWSKI_SCIENCE_2017_IN_CTX_MGE2_UPREG.html]

Details for gene set RICHARDS\_NOWAKOWSKI\_SCIENCE\_2017\_IN\_CTX\_MGE2\_UPREG[GSEA]

|  || Dataset | state23071 |
| Phenotype | NoPhenotypeAvailable |
| Upregulated in class | na\_neg |
| GeneSet | RICHARDS\_NOWAKOWSKI\_SCIENCE\_2017\_IN\_CTX\_MGE2\_UPREG |
| Enrichment Score (ES) | -0.50978184 |
| Normalized Enrichment Score (NES) | -2.9285812 |
| Nominal p-value | 0.0 |
| FDR q-value | 0.0 |
| FWER p-Value | 0.0 |
Table: GSEA Results Summary

  

Fig 1: Enrichment plot: RICHARDS\_NOWAKOWSKI\_SCIENCE\_2017\_IN\_CTX\_MGE2\_UPREG      
 Profile of the Running ES Score & Positions of GeneSet Members on the Rank Ordered List

  

| PROBE | GENE SYMBOL | GENE\_TITLE | RANK IN GENE LIST | RANK METRIC SCORE | RUNNING ES | CORE ENRICHMENT || 1 | NF2 |  |  | 103 | 1.036 | -0.0116 | No |
| 2 | KIF20B |  |  | 133 | 0.989 | -0.0052 | No |
| 3 | NUP85 |  |  | 328 | 0.729 | -0.0439 | No |
| 4 | TENM2 |  |  | 555 | 0.599 | -0.0924 | No |
| 5 | NETO2 |  |  | 740 | 0.519 | -0.1314 | No |
| 6 | RIMS1 |  |  | 776 | 0.506 | -0.1332 | No |
| 7 | NTN4 |  |  | 883 | 0.474 | -0.1533 | No |
| 8 | CASP2 |  |  | 884 | 0.474 | -0.1468 | No |
| 9 | SRSF2 |  |  | 941 | 0.461 | -0.1545 | No |
| 10 | ACKR3 |  |  | 962 | 0.454 | -0.1532 | No |
| 11 | NAE1 |  |  | 973 | 0.450 | -0.1495 | No |
| 12 | PDCD5 |  |  | 1051 | 0.428 | -0.1630 | No |
| 13 | TPRKB |  |  | 1168 | 0.403 | -0.1865 | No |
| 14 | LRRC8B |  |  | 1472 | 0.344 | -0.2579 | No |
| 15 | TRERF1 |  |  | 1530 | 0.335 | -0.2676 | No |
| 16 | CDC27 |  |  | 1562 | 0.330 | -0.2708 | No |
| 17 | FUS |  |  | 1571 | 0.329 | -0.2683 | No |
| 18 | ANKRD28 |  |  | 1635 | 0.319 | -0.2797 | No |
| 19 | TXLNA |  |  | 1646 | 0.317 | -0.2778 | No |
| 20 | CDC123 |  |  | 1647 | 0.317 | -0.2735 | No |
| 21 | PTBP3 |  |  | 1700 | 0.311 | -0.2822 | No |
| 22 | RAD23B |  |  | 1777 | 0.301 | -0.2972 | No |
| 23 | SLIT2 |  |  | 1873 | 0.289 | -0.3170 | No |
| 24 | PAK1IP1 |  |  | 1942 | 0.280 | -0.3302 | No |
| 25 | CDC5L |  |  | 1986 | 0.274 | -0.3373 | No |
| 26 | RNF34 |  |  | 2197 | 0.252 | -0.3865 | No |
| 27 | OCIAD2 |  |  | 2361 | -0.266 | -0.4238 | No |
| 28 | SEMA6D |  |  | 2407 | -0.272 | -0.4314 | No |
| 29 | SNX27 |  |  | 2435 | -0.275 | -0.4343 | No |
| 30 | RUNX1T1 |  |  | 2458 | -0.278 | -0.4360 | No |
| 31 | ANKRD50 |  |  | 2653 | -0.309 | -0.4805 | No |
| 32 | MIB1 |  |  | 2662 | -0.310 | -0.4782 | No |
| 33 | DCAF8 |  |  | 2679 | -0.313 | -0.4779 | No |
| 34 | ARRDC3 |  |  | 2684 | -0.314 | -0.4746 | No |
| 35 | CEP170 |  |  | 2737 | -0.324 | -0.4832 | No |
| 36 | SLC29A4 |  |  | 2741 | -0.325 | -0.4795 | No |
| 37 | OFD1 |  |  | 2754 | -0.327 | -0.4780 | No |
| 38 | KCTD12 |  |  | 2797 | -0.335 | -0.4839 | No |
| 39 | RNMT |  |  | 2805 | -0.336 | -0.4810 | No |
| 40 | SMARCC1 |  |  | 2837 | -0.342 | -0.4841 | No |
| 41 | ATCAY |  |  | 2865 | -0.346 | -0.4861 | No |
| 42 | TTC14 |  |  | 2867 | -0.347 | -0.4815 | No |
| 43 | GRIP2 |  |  | 2876 | -0.350 | -0.4787 | No |
| 44 | ADD1 |  |  | 2886 | -0.353 | -0.4761 | No |
| 45 | MSRA |  |  | 2891 | -0.354 | -0.4722 | No |
| 46 | TMEM59 |  |  | 2960 | -0.366 | -0.4843 | No |
| 47 | FIS1 |  |  | 3004 | -0.374 | -0.4899 | No |
| 48 | XRN1 |  |  | 3047 | -0.384 | -0.4951 | No |
| 49 | AKAP9 |  |  | 3066 | -0.389 | -0.4943 | No |
| 50 | KIAA1109 |  |  | 3116 | -0.403 | -0.5010 | No |
| 51 | ACOX1 |  |  | 3121 | -0.404 | -0.4965 | No |
| 52 | AGPAT4 |  |  | 3140 | -0.411 | -0.4953 | No |
| 53 | FAM110B |  |  | 3191 | -0.426 | -0.5020 | No |
| 54 | NARF |  |  | 3223 | -0.434 | -0.5038 | Yes |
| 55 | NPAS3 |  |  | 3233 | -0.437 | -0.5000 | Yes |
| 56 | TENM1 |  |  | 3235 | -0.437 | -0.4942 | Yes |
| 57 | NDUFC1 |  |  | 3244 | -0.438 | -0.4902 | Yes |
| 58 | STAU2 |  |  | 3269 | -0.445 | -0.4901 | Yes |
| 59 | PDZRN4 |  |  | 3279 | -0.450 | -0.4861 | Yes |
| 60 | HDAC9 |  |  | 3288 | -0.453 | -0.4819 | Yes |
| 61 | MEF2C |  |  | 3301 | -0.457 | -0.4786 | Yes |
| 62 | CREBBP |  |  | 3329 | -0.466 | -0.4789 | Yes |
| 63 | NMNAT2 |  |  | 3361 | -0.483 | -0.4800 | Yes |
| 64 | TMEM106B |  |  | 3402 | -0.499 | -0.4832 | Yes |
| 65 | SNRPN |  |  | 3403 | -0.499 | -0.4763 | Yes |
| 66 | ST8SIA4 |  |  | 3406 | -0.500 | -0.4699 | Yes |
| 67 | APC |  |  | 3422 | -0.510 | -0.4667 | Yes |
| 68 | JAKMIP2 |  |  | 3433 | -0.513 | -0.4621 | Yes |
| 69 | TAOK1 |  |  | 3442 | -0.516 | -0.4570 | Yes |
| 70 | ARX |  |  | 3443 | -0.516 | -0.4498 | Yes |
| 71 | SEMA6A |  |  | 3455 | -0.521 | -0.4454 | Yes |
| 72 | QKI |  |  | 3467 | -0.526 | -0.4409 | Yes |
| 73 | UQCR11 |  |  | 3469 | -0.527 | -0.4339 | Yes |
| 74 | SVIL |  |  | 3490 | -0.537 | -0.4315 | Yes |
| 75 | ATRX |  |  | 3509 | -0.546 | -0.4285 | Yes |
| 76 | BRK1 |  |  | 3564 | -0.580 | -0.4340 | Yes |
| 77 | ATXN1 |  |  | 3586 | -0.598 | -0.4310 | Yes |
| 78 | BBX |  |  | 3600 | -0.605 | -0.4260 | Yes |
| 79 | BLOC1S1 |  |  | 3630 | -0.627 | -0.4246 | Yes |
| 80 | CCNG2 |  |  | 3650 | -0.648 | -0.4204 | Yes |
| 81 | BCL11B |  |  | 3656 | -0.651 | -0.4127 | Yes |
| 82 | SERINC5 |  |  | 3676 | -0.664 | -0.4083 | Yes |
| 83 | NDUFB4 |  |  | 3678 | -0.668 | -0.3993 | Yes |
| 84 | PAM |  |  | 3685 | -0.671 | -0.3916 | Yes |
| 85 | MAGI2 |  |  | 3695 | -0.681 | -0.3844 | Yes |
| 86 | GAD1 |  |  | 3704 | -0.690 | -0.3769 | Yes |
| 87 | BMPR2 |  |  | 3715 | -0.698 | -0.3698 | Yes |
| 88 | ARID5B |  |  | 3723 | -0.701 | -0.3618 | Yes |
| 89 | ZNF91 |  |  | 3729 | -0.708 | -0.3533 | Yes |
| 90 | CXCR4 |  |  | 3733 | -0.710 | -0.3443 | Yes |
| 91 | NRXN3 |  |  | 3738 | -0.714 | -0.3354 | Yes |
| 92 | BCL11A |  |  | 3756 | -0.735 | -0.3295 | Yes |
| 93 | SATB1 |  |  | 3776 | -0.767 | -0.3237 | Yes |
| 94 | TNRC6B |  |  | 3781 | -0.771 | -0.3141 | Yes |
| 95 | GOLGB1 |  |  | 3795 | -0.785 | -0.3065 | Yes |
| 96 | BEX4 |  |  | 3796 | -0.785 | -0.2957 | Yes |
| 97 | SOX4 |  |  | 3797 | -0.786 | -0.2848 | Yes |
| 98 | MAPK10 |  |  | 3798 | -0.787 | -0.2740 | Yes |
| 99 | PFN2 |  |  | 3841 | -0.832 | -0.2730 | Yes |
| 100 | CCDC88A |  |  | 3854 | -0.848 | -0.2643 | Yes |
| 101 | SLAIN1 |  |  | 3862 | -0.855 | -0.2543 | Yes |
| 102 | ZEB2 |  |  | 3876 | -0.894 | -0.2452 | Yes |
| 103 | CADPS |  |  | 3877 | -0.894 | -0.2329 | Yes |
| 104 | MAF |  |  | 3893 | -0.930 | -0.2238 | Yes |
| 105 | SP9 |  |  | 3898 | -0.942 | -0.2118 | Yes |
| 106 | CUX1 |  |  | 3955 | -1.079 | -0.2110 | Yes |
| 107 | SOX6 |  |  | 3956 | -1.081 | -0.1960 | Yes |
| 108 | ZFHX4 |  |  | 3963 | -1.095 | -0.1824 | Yes |
| 109 | NCAM1 |  |  | 3966 | -1.106 | -0.1677 | Yes |
| 110 | SOX11 |  |  | 3977 | -1.147 | -0.1543 | Yes |
| 111 | TCF4 |  |  | 3984 | -1.173 | -0.1397 | Yes |
| 112 | VGLL4 |  |  | 3997 | -1.227 | -0.1257 | Yes |
| 113 | PTPRO |  |  | 4011 | -1.344 | -0.1104 | Yes |
| 114 | CHL1 |  |  | 4019 | -1.379 | -0.0932 | Yes |
| 115 | DLX1 |  |  | 4041 | -1.558 | -0.0769 | Yes |
| 116 | DLX2 |  |  | 4055 | -1.704 | -0.0567 | Yes |
| 117 | NRCAM |  |  | 4076 | -2.179 | -0.0316 | Yes |
| 118 | DCX |  |  | 4091 | -2.671 | 0.0018 | Yes |
Table: GSEA details [plain text format]

  

Fig 2: RICHARDS\_NOWAKOWSKI\_SCIENCE\_2017\_IN\_CTX\_MGE2\_UPREG: Random ES distribution      
 Gene set null distribution of ES for **RICHARDS\_NOWAKOWSKI\_SCIENCE\_2017\_IN\_CTX\_MGE2\_UPREG**
